# The Presence of Heat Shock Protein 70 (HSP70) Antibodies in Bilateral Meniere’s Disease: A Case Report

**DOI:** 10.7759/cureus.87843

**Published:** 2025-07-13

**Authors:** Stephanie Nagy, Marc M Kesselman

**Affiliations:** 1 Rheumatology, Dr. Kiran C. Patel College of Osteopathic Medicine, Nova Southeastern University, Davie, USA

**Keywords:** bilateral hearing loss, endolymphatic hydrops, heat shock protein-70, hsp70 antibodies, meniere’s disease

## Abstract

Meniere’s disease (MD) is a chronic, idiopathic inner ear disorder characterized by episodes of vertigo, fluctuating sensorineural hearing loss, tinnitus, aural fullness, gait disturbances, and postural instability. The pathophysiology is most commonly thought to be due to either an excess of endolymphatic fluid secretion or a failure to resorb the fluid into the subarachnoid space. The distension of the endolymphatic space disrupts the auditory and vestibular systems, leading to the symptoms experienced. Postulated causes of MD have been linked to allergies, genetic mutations, epigenetic factors, and alterations in anatomy, but an autoimmune cause is currently the strongest hypothesis. Specifically, the expansion of the endolymphatic space has been found to trigger the release of heat shock protein 70 (HSP70) antibodies, leading to MD. Although a causal link remains unconfirmed, these findings suggest an underlying autoinflammatory mechanism, warranting further investigation. We present a 29-year-old female who has bilateral MD, a rare finding in itself, in addition to being positive for HSP70 antibodies. This case underscores the importance of recognizing the potential autoimmune associations of MD, particularly in bilateral presentations, and contributes to the limited literature linking HSP70 antibodies to disease pathology and potential therapeutic targets.

## Introduction

Meniere’s disease (MD) is a chronic, debilitating, idiopathic disorder of the vestibular system. The cardinal symptoms include vertigo, hearing loss, tinnitus, and pressure within the ear. Less commonly, patients have complained about fullness in the ear, walking disturbances, postural instability, Tumarkin's otolithic crisis, also known as drop attacks, and nausea [1]. It was first described by Prosper Meniere in the 1860s, who identified that the symptoms of this condition were due to an inner ear pathology, rather than what was previously thought to occur due to “cerebral congestion.” Following this discovery in 1937, researchers identified endolymphatic hydrops as a cause for MD, as it results in the swelling of the membranous labyrinth, distending the endolymphatic space of the inner ear [2].

The prevalence of MD greatly varies worldwide. Reported prevalence rates of MD range from 3.5 per 100,000 to 513 per 100,000 [3-5]. This range has been found to be due to the rarity of MD. Specifically, in the United States, the prevalence was found to be 190 out of every 100,000 people. MD is found to be more common in females than males overall, with ratios ranging from 1.57 to 4.3:1 [3,5,6]. It is found to affect those between the ages of 40 and 70, with a peak of onset between 40 and 50 years old [7,8]. MD impacts predominantly adults, with only 2.3% of cases occurring in those under 18 [9]. The prevalence increases with age, rising from 61 per 100,000 among patients aged 18 to 34 to 440 per 100,000 in those over 65 years [6]. Additionally, those of the Caucasian race were found to develop MD at a two-fold increase [8].

The pathophysiology is due to either an excess of endolymphatic fluid secretion or a failure to resorb the fluid into the subarachnoid space. The distension of the endolymphatic space disrupts the auditory and vestibular systems [10]. Postulated causes behind the development of MD have been linked to a variety of triggers, including alterations in anatomy, allergies, genetic mutations, and autoimmune causes [11]. A wide variety of anatomy variations have been investigated as potential causes of MD, including obstructed endolymphatic ducts, thickening of the round window, decreased thickness of the otolithic membrane, alternation of the epithelial and mesothelial cells in the Reissner’s membrane, loss of spiral ganglion or cochlear hair cells, and thinning of the stria vascularis (lateral wall of the cochlea) [12]. In terms of allergies, it is thought that the inner ear may be susceptible to allergic reactions, as elevated IgE, interleukin (IL)-4, IL-5, IL-10, and IL-13 were noted [13]. A strong family history has been linked to MD. It usually follows an autosomal dominant inheritance pattern with reduced penetrance, indicating that family members will display varying symptoms [14,15]. Within familial MD, genes associated with the structure and function of the inner ear have been found to be mutated between family members, including the OTOG, MYO7A, TECTA, FAM136A, DTNA, PRKCB, SEMA3D, and DPT genes [14,15]. However, the autoimmune hypothesis is currently the strongest. Ideas surround the upregulation of the NLRP3 inflammasome within macrophages, triggering IL-1 beta, which has been linked to sensorineural hearing loss; also, the elevation of other pro-inflammatory cytokines have been found, including tumor necrosis factor-alpha (TNF-α), IL-1 alpha, cutaneous T-cell attracting chemokine (CTACK), macrophage inflammatory protein-1 alpha (MIP-1α), and macrophage inflammatory protein-1 beta (MIP-1β) [11,16].

But, recently, there has been an investigation into the link between autoinflammatory antibodies and MD, which seems to be proven as the strongest autoimmune link. Specifically, the relationship between heat shock protein 70 (HSP70) antibodies and MD. HSP70 is a chaperone protein that maintains proper protein homeostasis and is essential for cellular functioning during stressful conditions. It assists in the proper folding of proteins, refolding of denatured proteins, protein transport, and stabilization. They also have a significant role in the immune system by enhancing both adaptive and innate immune responses. HSP70 is able to bind to antigen-presenting cells to activate T-cell responses, and they are also able to upregulate pro-inflammatory cytokines that lend it to trigger autoimmune reactions [17]. Autoantibodies to HSP70 have already been linked to other autoimmune conditions such as rheumatoid arthritis, celiac disease, juvenile idiopathic arthritis, and autoimmune liver disease [18-21].

There are limited case studies in the literature that explore the association between HSP70 antibodies and MD. This paper presents the case of a 29-year-old female diagnosed with bilateral MD who tested positive for HSP70 antibodies. This case contributes to the growing body of evidence supporting a potential autoinflammatory mechanism in the pathogenesis of MD.

## Case presentation

The patient was a 29-year-old female presenting to the rheumatology outpatient clinic with telangiectasia, polyarthralgia, chronic urticaria, and previously diagnosed bilateral MD via another provider. The patient had experienced vertigo, dizziness, and falls. She noted previous symptoms of hives, facial telangiectasia, pruritus, photosensitivity, and difficulty using her hands to open and close doors, jars, and other objects. She stated she was diagnosed by previous providers with chronic urticaria, positive antinuclear antibodies (ANA), hypermobility, and an allergic disorder. She was prescribed omalizumab by previous providers for chronic urticaria. There was a family history of undisclosed thyroid problems, gout, colon cancer, and Crohn’s disease. Past medical history included polycystic ovarian syndrome, migraines, parotid tumor removal, and Sicca syndrome (a condition classified by dry eyes and mouth).

In the patient’s review of systems, she denied any chills, fatigue, fever, malaise, night sweats, weight gain, weight loss, nasal or ear drainage, hearing loss, sinus pressure, sore throat, tinnitus, vertigo, eye pain, eye discharge, vision loss, vision changes, cough, dyspnea, known tuberculosis exposure, chest pain, edema, palpitations, abdominal pain, change in bowel movements, nausea, vomiting, dysuria, hematuria, polyuria, incontinence, polydipsia, polyphagia, dizziness, weakness, headache, numbness, syncope, seizures, tremors, anxiety, depression, insomnia, brittle hair or nails, back pain, easy bruising or bleeding, and lymphadenopathy. She was positive for pruritus, rash, urticaria, joint pain, and bilateral hand pain. The patient has had no recent sick contacts or recent travel. Her current medications included Lexapro 10 mg and 20 mg orally (PO) daily, Qulipta 60 mg PO daily, vitamin D3 50 mg capsule PO daily, Pepcid 20 mg PO twice a day (BID), Allegra 24 hour release 180-240 mg tablet PO daily, montelukast 10 mg PO daily, Xyzal 5 mg PO daily, tizanidine 4 mg as needed (PRN) every six to eight hours, and buspirone 5 mg PO BID. The patient was allergic to non-steroidal anti-inflammatory drugs (NSAIDs), and the reaction involved abdominal discomfort. The most recent laboratory results are shown in Tables 1-3.

**Table 1:** Laboratory results for antibodies.

Labs	Findings	Normal range
Antinuclear antibody	Positive	Titer <1:40
B2 glycoprotein (IgA, IgG, IgM)	Negative <2.0	<20 U/ml
Cardiolipin (IgA, IgG, IgM)	Negative <2.0	<12.5 U/ml IgM, <15 U/ml IgG
Centromere B antibody	Negative <0.1	<0.1 antibody index
Anti-chromatin antibody	Negative <0.1	0-20 units
Cyclic citrullinated protein	Negative <16	<20 EU/mL
Double-stranded DNA antibody	Negative	<10 IU/mL
Jo-1 antibody	Negative <0.1	0 units
Anti-aminoacyl tRNA synthetase antibody (EJ antibody)	Negative <11	Negative
Anti-melanoma differentiation-associated gene 5 antibody	Negative <11	<20 units
Anti-nuclear matrix protein 2 - alpha and beta antibody	Negative <11	Negative
Nuclear matrix protein 2 antibody	Negative <11	<20 units
Anti-isoleucyl-tRNA synthetase antibody	Negative <11	Negative
Anti-alanyl-tRNA synthetase antibody	Negative <11	Negative
Anti-threonyl-tRNA synthetase antibodies antibody	Negative <11	Negative
Signal recognition particle antibody	Negative <11	Negative
Rheumatoid factor	Negative <5	<20 U/ml
Ribonucleoprotein antibody	Negative <0.1	<1 unit
Scleroderma antibody-70	Positive 1.7	<1 unit
Sjögren’s antibody	Negative <0.1	<1 unit/ml
Anti-Smith antibody	Negative <0.1	<7 unit/ml
Antineutrophil cytoplasmic antibody	Negative	Titer <1:20
Myeloperoxidase antibody	Negative <0.1	0-0.9 antibody index
Proteinase-3 antibody	Negative <0.1	<0.4 Units
Angiotensin-1-converting enzyme	Positive 45	<40 mcg/L
HIV antigen/antibody 4^th^ generation	Non-reactive	Non-reactive
HLA-B27 antigen	Negative	Negative
Heat shock protein 70	Positive	Negative
Lyme antibody 18KD (IgG) band	Non-reactive	Non-reactive
Lyme antibody 23KD (IgG) band	Non-reactive	Non-reactive
Lyme antibody 23KD (IgM) band	Non-reactive	Non-reactive
Lyme antibody 28KD (IgG) band	Non-reactive	Non-reactive
Lyme antibody 30KD (IgG) band	Non-reactive	Non-reactive
Lyme antibody 39KD (IgG) band	Non-reactive	Non-reactive
Lyme antibody 39KD (IgM) band	Reactive	Non-reactive
Lyme antibody 41KD (IgG) band	Reactive	Non-reactive
Lyme antibody 41KD (IgM) band	Non-reactive	Non-reactive
Lyme antibody 45KD (IgG) band	Non-reactive	Non-reactive
Lyme antibody 58KD (IgG) band	Reactive	Non-reactive
Lyme antibody 66KD (IgG) band	Non-reactive	Non-reactive
Lyme antibody 93KD (IgG) band	Reactive	Non-reactive
Lyme disease antibody IgG	Negative	Negative
Lyme disease antibody IgM	Negative	Negative
Saccharomyces cerevisiae IgG antibody	Negative 6.5	<20 units
Saccharomyces cerevisiae IgA antibody	Negative 15.8	<20 units

**Table 2:** Laboratory results for complete blood count.

Labs	Findings	Normal range
Mean platelet volume	9.4	7.5-11.5 fl
Mean corpuscular hemoglobin	29.9	27-31 pg/cell
Mean corpuscular volume	91.2	80-100 fl
Basophils	0.4	0-1%
Eosinophils	1.1	0-3%
Lymphocytes	42.1	24-44%
Hematocrit	39.3	42-52%
Hemoglobin	12.9	14-18 g/dL
Erythrocyte sedimentation rate	2	<20 mm/hr

**Table 3:** Laboratory results for complete metabolic panel.

Labs	Findings	Normal range
Calcium	9.5	8.5-10.2 mg/dL
Carbon dioxide	25	23-29 mEq/L
Chloride	106	96-106 mEq/L
Creatinine	0.61	0.6-1.3 mg/dL
Estimated glomerular filtration rate	124	60-89 ml/min/1.73 m^2^

Of significance, the patient was positive for scleroderma antibody-70 (SCL-70) antibodies, ANA, HSP70 antibodies, four Lyme bands (Lyme antibody 39KD (IgM) band, Lyme antibody 41KD (IgG) band, Lyme antibody 58KD (IgG) band, and Lyme antibody 93KD (IgG) band), and angiotensin-1-converting enzyme.

A thorough musculoskeletal exam was conducted on all four extremities, with no abnormal findings. The presence of SCL-70 antibodies in conjunction with the patient’s polyarthralgia indicates a potential inflammatory cause; however, a well-known side effect of omalizumab, which the patient was prescribed previously for her chronic urticaria, is polyarthralgia. It was recommended to halt omalizumab as a trial to see if the bilateral hand pain improved. The patient was referred for additional blood tests to evaluate antineutrophil cytoplasmic antibody (ANCA) titers, C-reactive protein levels, complete blood count, and a comprehensive metabolic panel. The patient was positive for four Lyme bands; however, she did not have any recent travel and did not recall a tick exposure. Lyme disease can cause neurological and sensory disturbances like vertigo and hearing loss; however, with the patient's history and lack of additional Lyme disease findings, this is a less likely etiology. Positive angiotensin-1-converting enzyme is usually associated with sarcoidosis; however, the patient has no additional common associated findings. The patient was also referred back to ENT to further examine the patient's MD following positive HSP70 antibodies.

Focusing on bilateral MD with positive HSP70 antibodies is particularly significant, given the limited evidence available in the current literature. Only a few case studies have reported this association, and bilateral MD alone is a rare clinical finding; its co-occurrence with HSP70 antibody positivity is even more uncommon. HSP70 antibodies have previously been implicated in autoimmune hearing loss, and expanding the literature in this area may offer further support for an autoinflammatory etiology in the pathogenesis of MD.

## Discussion

MD is a chronic condition that is diagnosed based on exclusion. Patients commonly present with vertigo, dizziness, hearing loss, tinnitus, and pressure in the ear, thought to be due to an increase in fluid in the endolymphatic space. MD most commonly occurs unilaterally, rarely bilaterally [1,22].

This diagnosis of MD is based on criteria determined by the American Academy of Otolaryngology-Head and Neck Surgery. They determined two criteria, one for definitive MD and the other for probable MD. Definitive MD diagnosis is based on a patient having two or more spontaneous vertigo attacks lasting between 20 minutes and 12 hours, audiometrically determined low to mid frequency sensorineural hearing loss in the affected ear(s) on at least one occurrence before, during or after the vertigo episode, with aural symptoms in the affected ear(s), and all other possible causes being excluded. Probable MD diagnosis is based on the patient having at least two episodes of vertigo or dizziness lasting between 20 minutes and 24 hours, with aural symptoms in the affected ear(s), and other causes being excluded [23]. The Meniere’s Disease Consortium further identified five subtypes of MD. Type 1 was the most common type with unilateral MD hearing loss without migraine or autoimmune disease. Type 2 included patients with vertigo episodes, followed by delayed hearing loss that occurred months to years later without migraines or autoimmune disease. Type 3 is classified as familial MD, with a hypothesized genetic origin. Type 4 is any presence of migraines with MD. Finally, type 5 is those with autoimmune conditions in addition to MD. It was found that autoimmune disorders occur more commonly in those with bilateral MD. This directly links to our patient case, with bilateral MD with a high suspicion of scleroderma [24,25]. Differential diagnoses to rule out prior to diagnosing patients with MD include basilar migraines, vestibular neuronitis, benign paroxysmal positional vertigo, central vertigo, peripheral vertigo, vestibular schwannomas, meningioma, and meningitis, to name a few [1].

**Figure 1:**
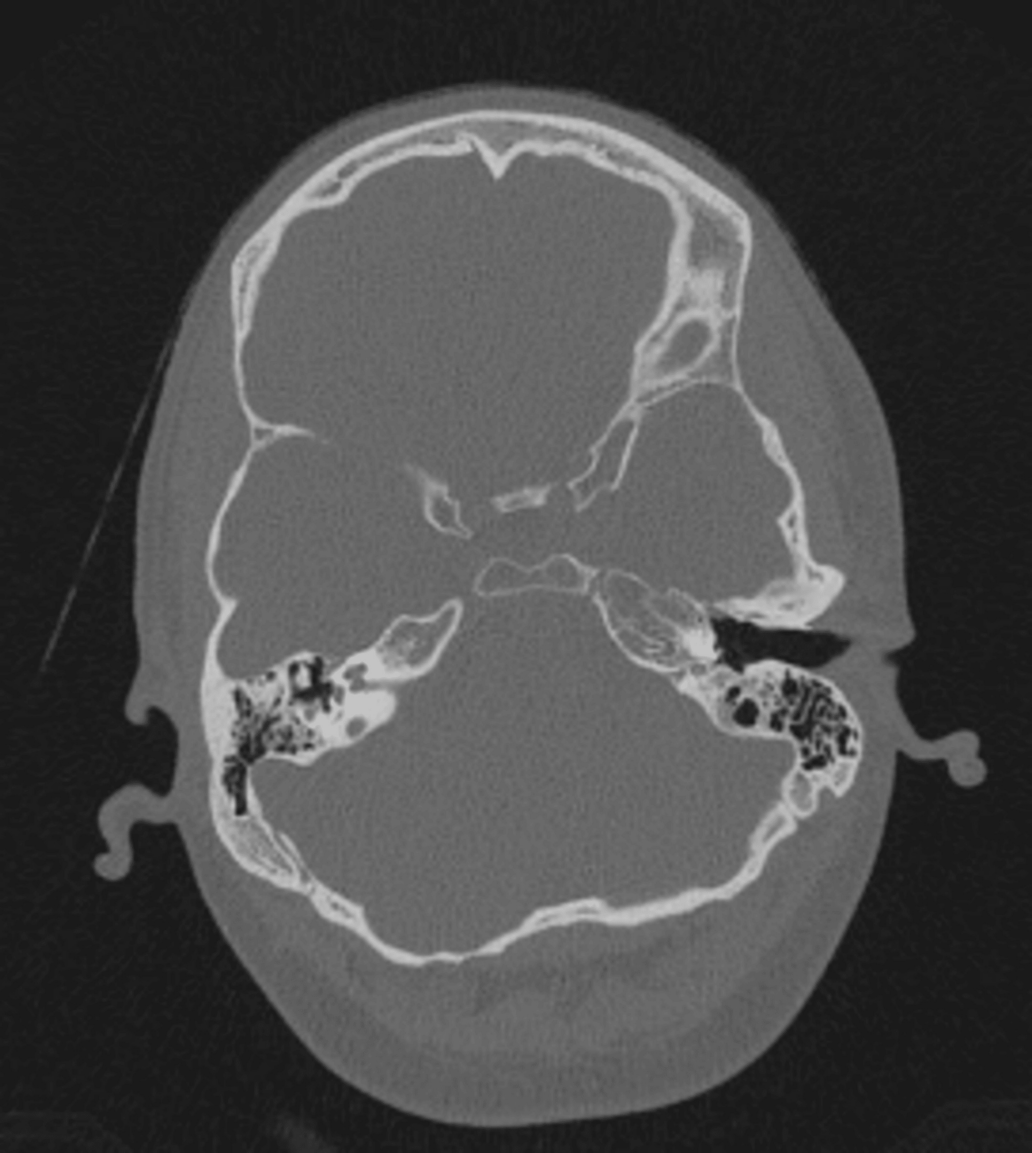
Axial CT in bone window indicating bilateral endolymphatic hydrops. Source: Gerstenmaier J. Ménière disease. Radiopaedia.org. (Accessed on 09 May 2025). rID: 24268 (https://doi.org/10.53347/rID-24268). Used under the Creative Commons Attribution-NonCommercial-ShareAlike license.

**Figure 2:**
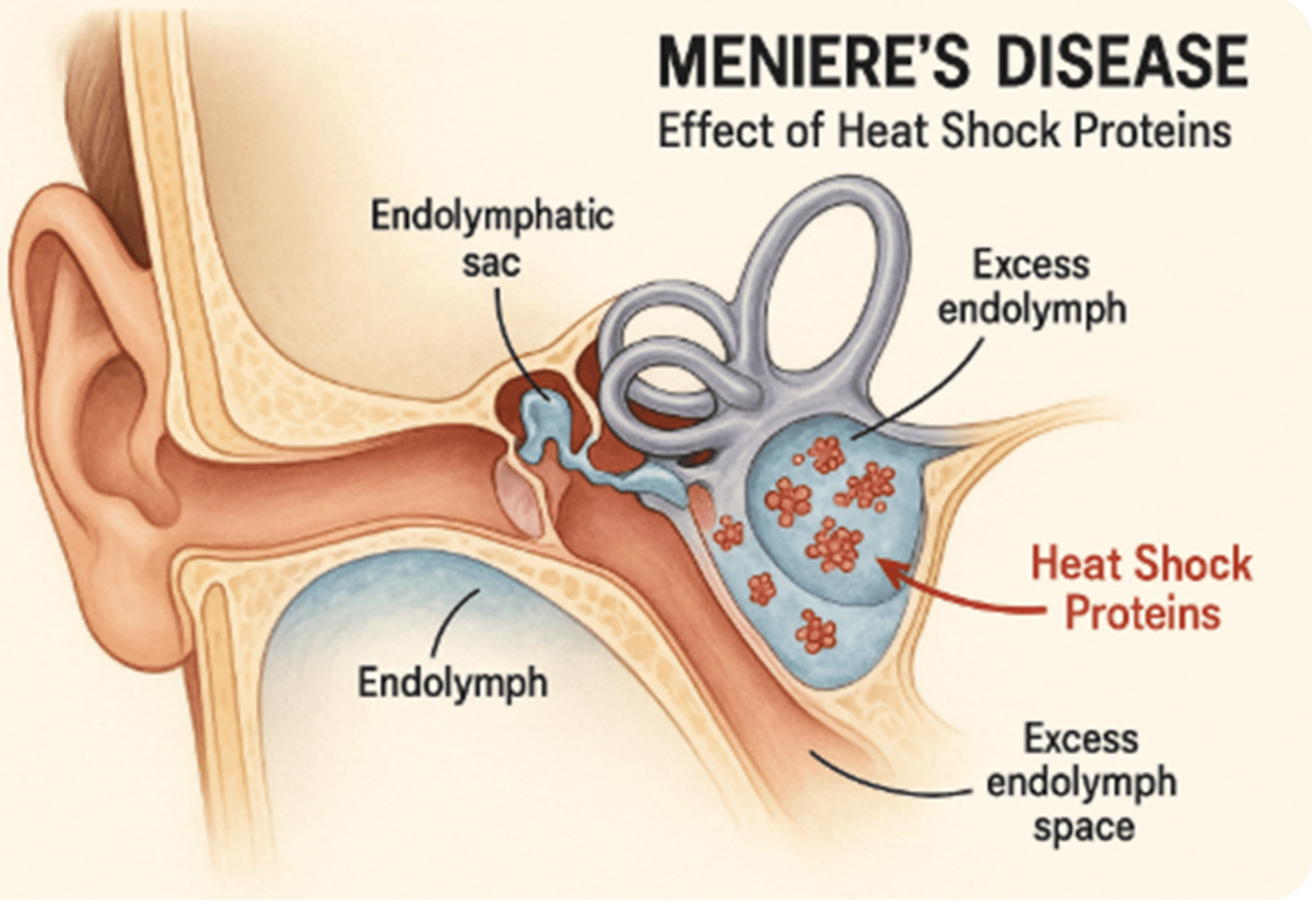
The diagram highlights the cochlea and vestibular structures, illustrating the area affected by fluid accumulation within the endolymphatic space. Image generated using ChatGPT (OpenAI, San Francisco, CA), from the prompt endolymphatic hydrops due to heat shock proteins.

As mentioned, various etiological causes of MD have been hypothesized, including autoimmune conditions, allergies, genetic mutations, epigenetic factors, and alterations in anatomy. The leading hypothesis indicates an autoimmune process. It has been recommended that patients who present with any autoimmune condition plus fulfill criteria for MD should be subsequently diagnosed with autoimmune MD. It has been found that those with MD are at a three- to eight-fold increased risk of also developing an autoimmune condition [8,11,26]. The theories surrounding the autoimmune cause of MD include cross-reactivity of antibodies to T-cells, cytokine damage, and reaction against self-antigens in the ear [27,28]. One of the leading antibodies that has been investigated is HSP70. Hornibrook et al. found that 17.5% of those diagnosed with MD were positive for HSP70 antibodies [29]. In another study by Shin et al., they found that 33% of patients with MD had HSP70 antibodies versus only 5% in a control group [30]. Even more interestingly, HSP70 antibodies have been found more commonly in bilateral MD, which is seen in this patient case. HSP70 antibodies were seen to be positive in 58.8% of those with bilateral MD compared to only 33.3% in those with unilateral disease [31]. The reason behind the presence of HSP70 antibodies in MD is still under discovery. It is thought that the inflammation of the inner ear due to endolymphatic hydrops may trigger the release of HSP70 antibodies due to the distention of the ear (Figures 1, 2). The question still remains if there are certain patient characteristics that result in positive HSP70 antibodies in some patients with MD, especially those with bilateral MD. Additionally, could HSP70 be a target for future medications in those who are found to have these antibodies? There has also been a lack of case studies reporting these findings in the literature; as a result, we would like to emphasize this connection within this patient report.

To diagnose MD, an audiometric evaluation is required to assess hearing loss, as low-frequency sensorineural hearing loss is a classic finding. It is then recommended that these patients undergo an MRI to rule out other cochlear abnormalities and indicate endolymphatic hydrops [32,33]. 3D maximum intensity projections were seen to evaluate endolymphatic hydrops better than 2D images on MRI [34]. Injecting gadolinium into the tympanic membrane can help visualize the presence of endolymphatic hydrops [35].

There is no definitive treatment for MD. Diet recommendations for those with MD include limiting salt intake because it can prevent MD attacks by elevating aldosterone levels, which can lead to the absorption of sodium and the endolymphatic fluid. Patients on low-sodium diets reported improvements in hearing, vertigo, and tinnitus [36-38]. Thiazide diuretics have also been investigated in the treatment of MD by reducing fluid in the inner ear to reduce symptoms [36]. A new medication, betahistine dihydrochloride, has been found to reduce vertigo attacks, but there was no significant difference when compared to a placebo [39]. More invasive options for MD include intratympanic steroid injections, which reduce vertigo attacks, and intratympanic gentamicin injections, which reduce vertigo and aural fullness; however, they cause elevated hearing loss due to being ablative to vestibular cells [40,41].

Overall, MD is not a cause of mortality. The overall disease progression is still debated. It has been found that patients experience elevated episodes of vertigo in the first few years of disease; however, the episodes decrease with time, regardless of the treatment received. It was found that 70% of those who have been in remission for over a year generally continue to be episode-free [42]. However, oppositely, Havia et al. found that those with MD less than 10 years had a lower incidence of vertigo and less severe episodes compared to those with more than 20 years of MD [22]. The risk of hearing loss (approximately 50-60 dB) was also found to be elevated in the first few years of the disease. Interestingly, those with unilateral MD are found to be at greater risk of hearing loss compared to those with bilateral MD [43,44].

## Conclusions

This patient case report contributes valuable insight to the limited existing literature on MD, particularly highlighting a rare presentation of bilateral involvement with positive HSP70 antibodies. The presence of these antibodies further supports a potential autoimmune component in the pathophysiology of MD. By documenting this unique clinical profile, the report underscores the need for continued investigation into the immunologic mechanisms that may underlie MD. In particular, future research should explore the role of HSP70 antibodies in the disease’s etiology, progression, and symptomatology. A deeper understanding in this area may pave the way for novel diagnostic markers and targeted immunomodulatory therapies, ultimately improving patient outcomes.
